# Supplementary material for: A Novel AURKA Mutant-Induced Early-Onset Severe Hepatocarcinogenesis Greater than Wild-Type via Activating Different Pathways in Zebrafish
Source: Cancers (Basel). 2019 Jul 2;11(7):927. doi: 10.3390/cancers11070927 (PMC6678475; doi:10.3390/cancers11070927)
Supplement: Supplementary file 1 [file cancers-11-00927-s001.pdf]

Supplementary Material

A Novel AURKA Mutant-Induced Early-Onset Severe Hepatocarcinogenesis Greater than Wild-Type via Activating Different Pathways in Zebrafish

Zhong-Liang Su Chien-Wei Su, Yi-Luen Huang, Wan-Yu Yang, Bonifasius Putera Sampurna, Toru Ouchi, Kuan-Lin Lee, Chen-Sheng Wu, Horng-Dar Wang and Chiou-Hwa Yuh

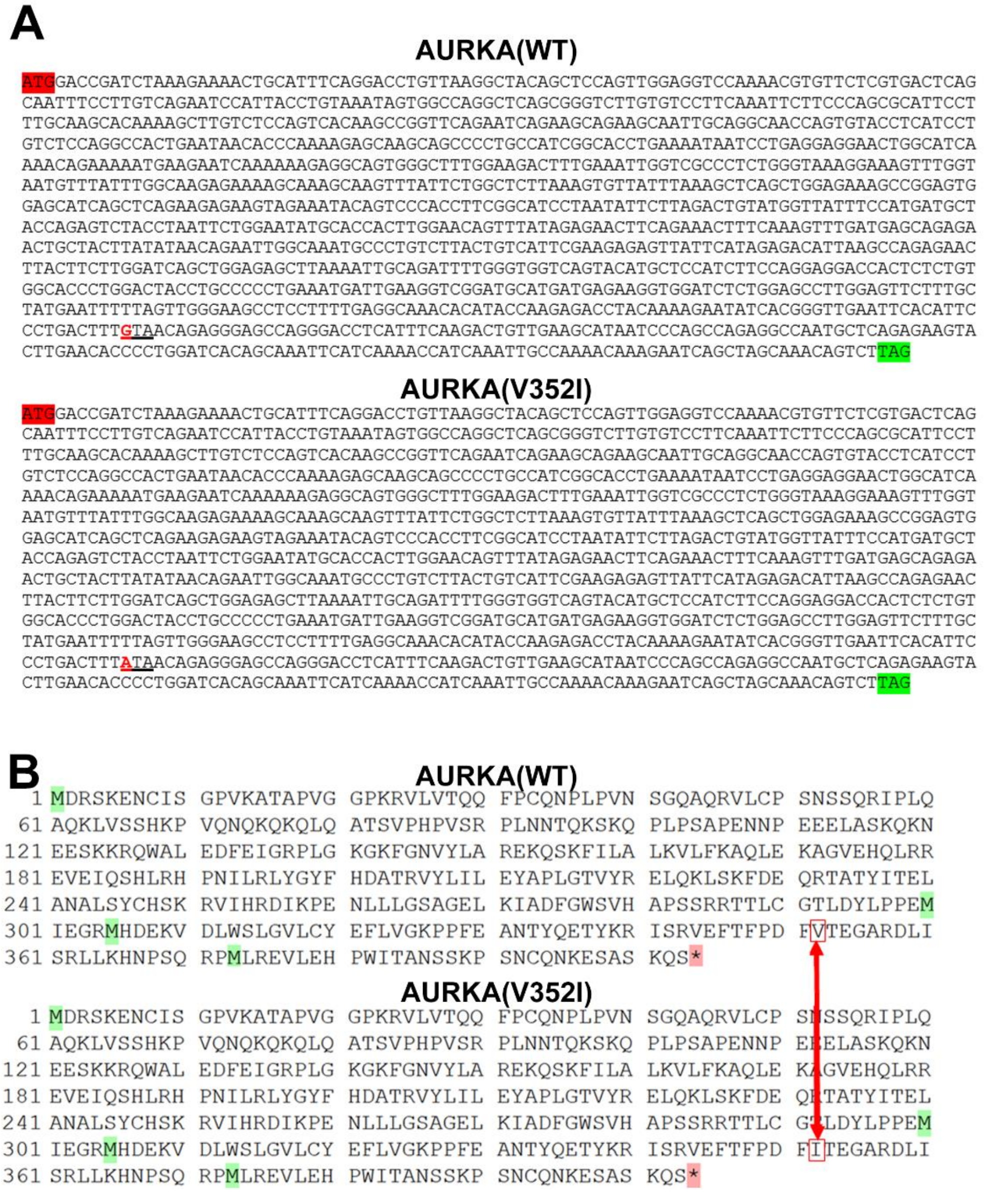

Figure S1. Sequence of AURKA (WT) and AURKA (V352I). (**A**) mRNA sequence of AURKA (WT) and AURKA (V352I), the red indicates the mutated nucleotide sequence. (**B**) Protein sequence of AURKA (WT) and AURKA (V352I), the red box indicates the mutated amino acid sequence.

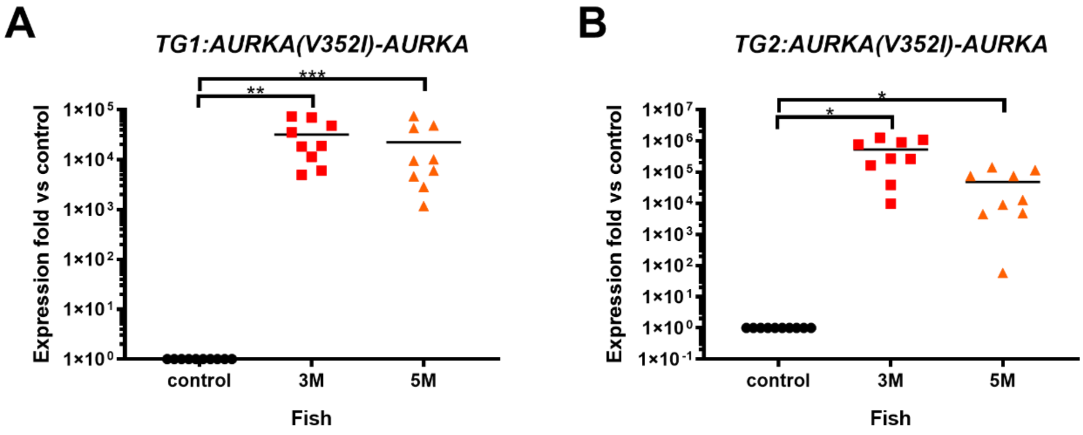

Figure S2. Expression of AURKA in TG1-AURKA(V352I) and TG2-AURKA(V352I) transgenic zebrafish compared with control fish. qPCR analysis of AURKA in TG1-AURKA(V352I) and TG2-AURKA(V352I) transgenic zebrafish compared to control fish at 3 M and 5 M. (**A**) TG1: Tg(fabp10a:AURKA(V352I)-EGFP-mCherry, myl7:EGFP). (**B**) TG2: Tg(fabp10a:AURKA(V352I)-EGFP- mCherry, myl7:EGFP). Expression fold compared to control fish (Tg(fabp10a: EGFP- mCherry) was shown in red (3M) and orange (5M). Statistical analysis of results was performed using a two-tailed Student's *t*-test. Asterisks (*) represent the level of significance. *: p-value ≤0.05; **: p-value ≤ 0.01; ***: p-value ≤ 0.001.

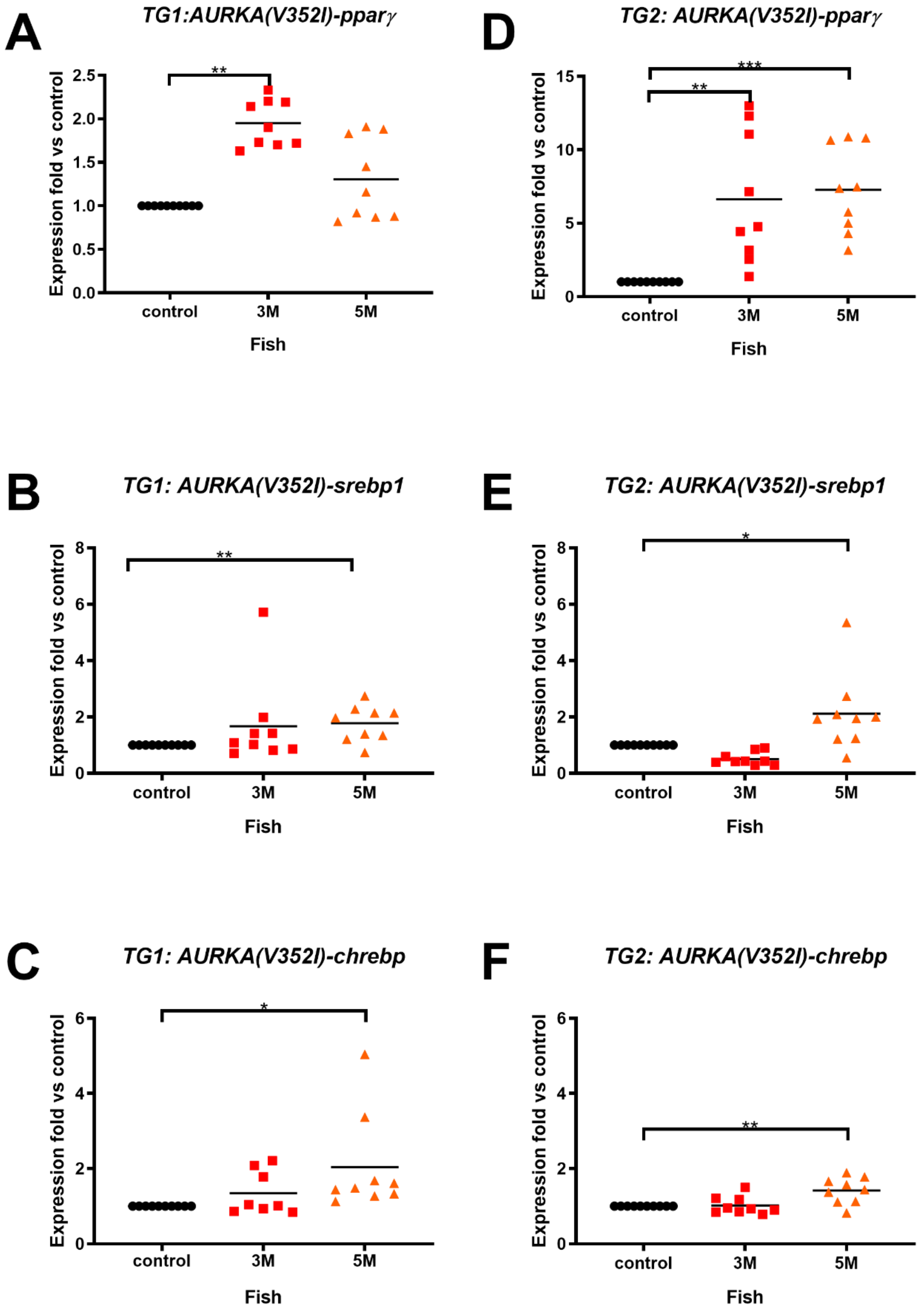

Figure S3. Expression of lipogenic factors (*pparγ, srebp1, chrebp*) in TG1-AURKA(V352I) and TG2-AURKA(V352I) transgenic fish were higher than control. qPCR analysis of lipogenic factors (**A, D**) *pparγ;* (**B, E**) *srebp1;* (**C, F**) *chrebp* in TG1-AURKA(V352I) and TG2-AURKA(V352I) transgenic zebrafish compared to control fish at different time points. Statistical analysis of results was performed using a two-tailed Student's *t*-test. Asterisks (*) represent the level of significance. *: *p*-value≤0.05; **: *p*-value ≤ 0.01; ***: *p*-value ≤ 0.001.

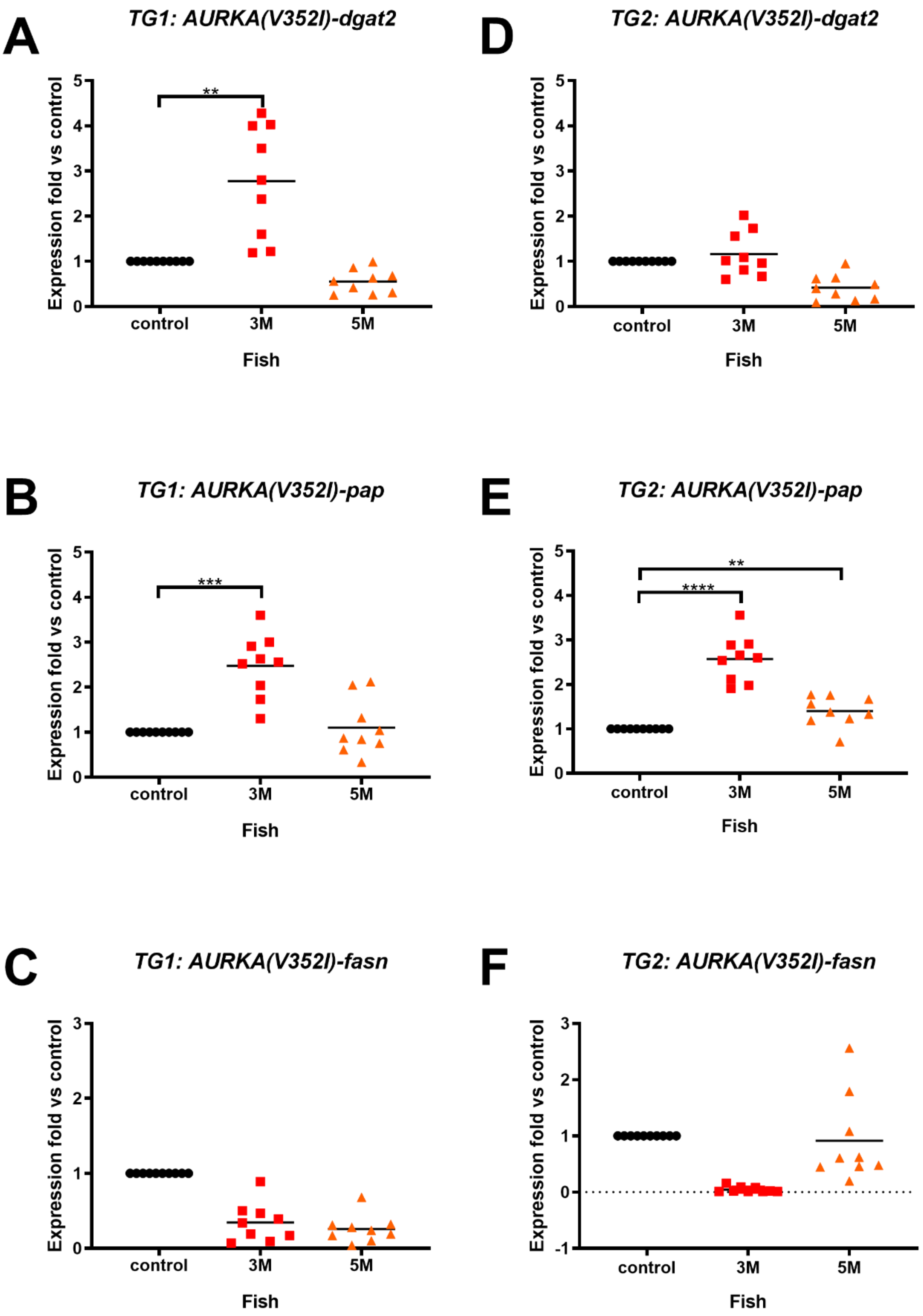

Figure S4. Expression of lipogenic enzymes (*dgat2*, *pap*, *fasn*) in TG1-AURKA (V352I) and TG2-AURKA (V352I) transgenic fish were higher than control. qPCR analysis of lipogenic enzyme (**A, D**) *dgat2*; (**B, E**) *pap*; (**C, F**) *fasn* in TG1-AURKA(V352I) and TG2-AURKA(V352I) transgenic zebrafish compared to control fish at different time points. Statistical analysis of results was performed using a two-tailed Student's *t*-test. Asterisks (*) represent the level of significance. **: p-value ≤ 0.01; ***: p-value ≤ 0.001.

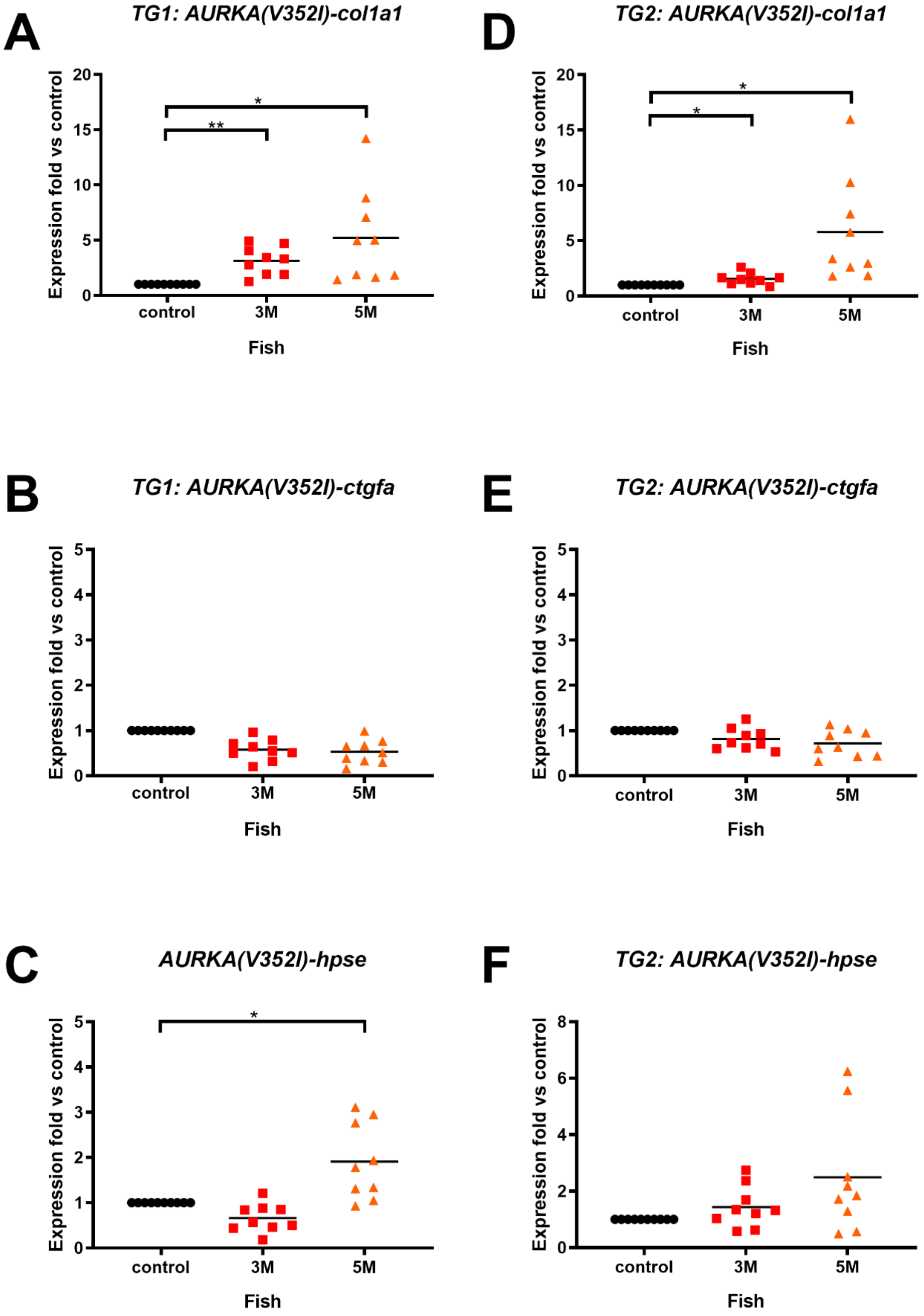

Figure S5. Expression of fibrosis markers (*col1a1*, *ctgfa*, *hpse*) in TG1-AURKA (V352I) and TG2-AURKA (V352I) transgenic fish compared to control. qPCR analysis of fibrosis markers (**A, D**) *col1a1*; (**B, E**) *ctgfa*; (**C, F**) *hpse* in TG1-AURKA(V352I) and TG2-AURKA(V352I) transgenic zebrafish compared to control fish at different time points. Statistical analysis of results was performed using a two-tailed Student's *t*-test. Asterisks (*) represent the level of significance. *: p-value ≤ 0.05; **: p-value ≤ 0.01.

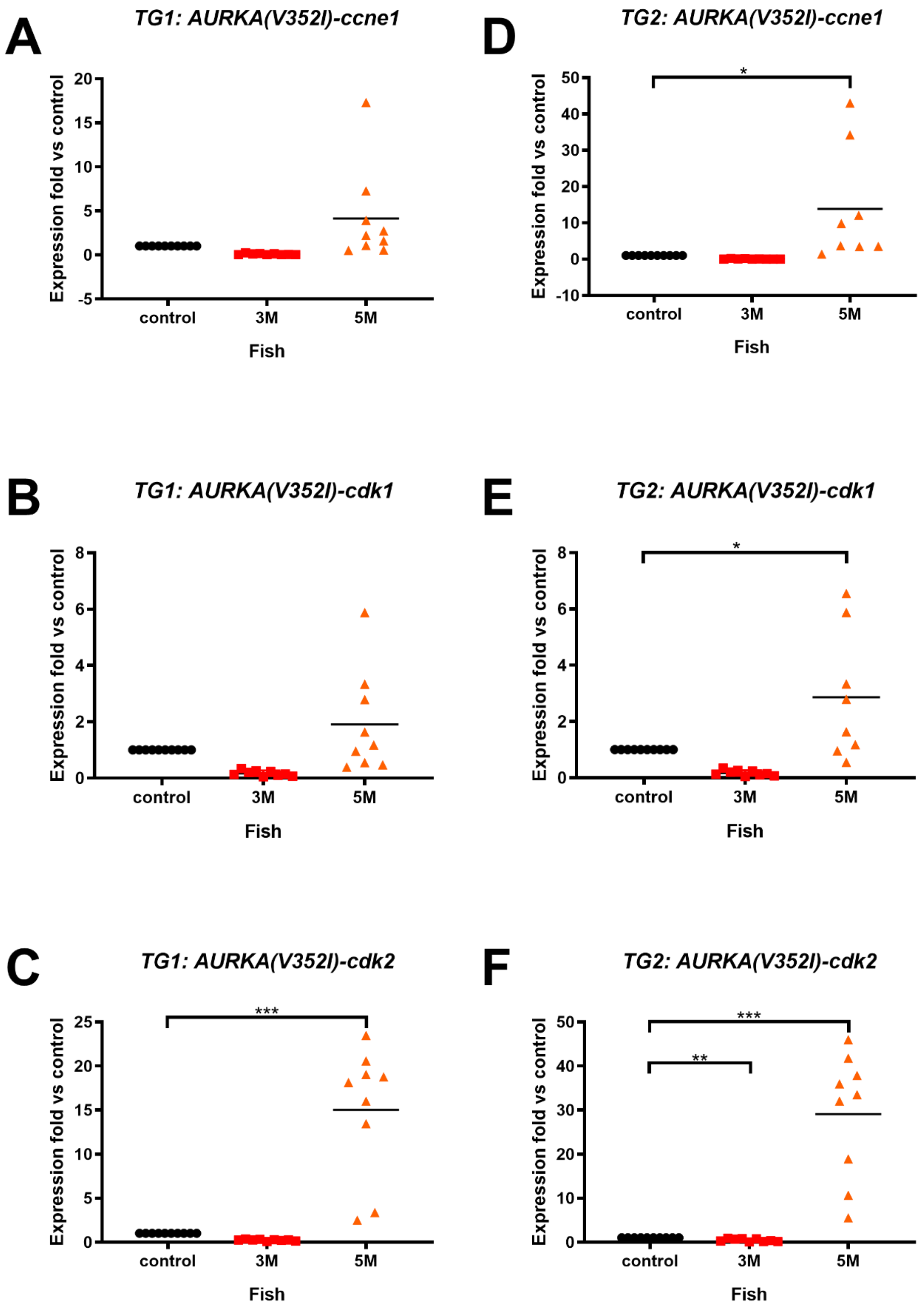

Figure S6. Expression of cell cycle related genes (*ccne1*, *cdk1*, *cdk2*) was higher in TG1-AURKA (V352I) and TG2-AURKA (V352I) transgenic fish. qPCR analysis of cell cycle/proliferation markers (**A, D**) *ccne1*; (**B, E**) *cdk1*; (**C, F**) *cdk2* in TG1-AURKA(V352I) and TG2-AURKA(V352I) transgenic zebrafish compared to control fish at different time points. Statistical analysis of results was performed using a two-tailed Student's *t*-test. Asterisks (*) represent the level of significance. *: p-value ≤ 0.05; **: p-value≤0.01; ***: p-value ≤ 0.001.

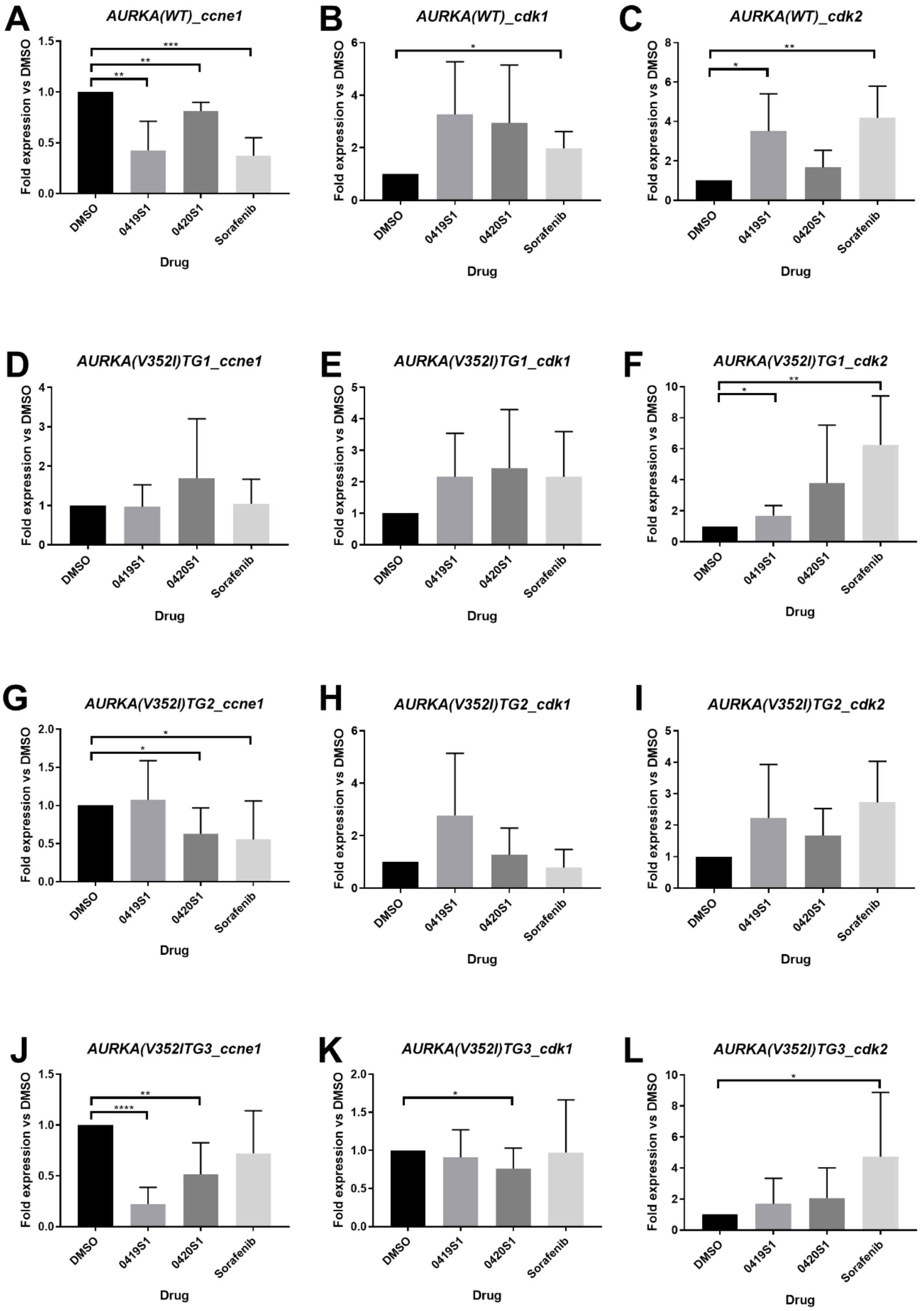

Figure S7. Expression of cell cycle related genes (*ccne1*, *cdk1*, *cdk2*) in AURKA(WT), TG1-AURKA(V352I), TG2-AURKA(V352I), and TG3-AURKA(V352I) transgenic fish after oral feeding of 0419S1, 0420S1 and Sorafenib for one month. qPCR analysis of cell cycle/proliferation markers (**A, D, G, J**) *ccne1*; (**B, E, H, K**) *cdk1*; (**C, F, I, L**) *cdk2* in AURKA (WT), TG1-AURKA(V352I), TG2-AURKA(V352I), and TG3-AURKA (V352I) transgenic zebrafish compared to control fish at different time points. Statistical analysis of results was performed using a two-tailed Student's *t*-test. Asterisks

(*) represent the level of significance. *: p-value ≤ 0.05; **: p-value ≤ 0.01; ***: p-value ≤ 0.001; ****: p-value ≤ 0.0001.
